# Similar cost of *Hamiltonella defensa* in experimental and natural aphid‐endosymbiont associations

**DOI:** 10.1002/ece3.8551

**Published:** 2022-01-24

**Authors:** Heidi Kaech, Stephanie Jud, Christoph Vorburger

**Affiliations:** ^1^ Eawag, Swiss Federal Institute of Aquatic Science and Technology Dübendorf Switzerland; ^2^ D‐USYS, Department of Environmental Systems Science ETH Zürich Zürich Switzerland

**Keywords:** *Aphis fabae*, cost of resistance, defensive symbiosis, endosymbiont, fitness, *Hamiltonella defensa*

## Abstract

Endosymbiont‐conferred resistance to parasitoids is common in aphids, but comes at a cost to the host in the absence of parasitoids. In black bean aphids (*Aphis fabae*), costs in terms of reduced lifespan and lifetime reproduction were demonstrated by introducing 11 isolates of the protective symbiont *Hamiltonella defensa* into previously uninfected aphid clones. Transfection of *H*.* defensa* isolates into a common genetic background allows to compare the costs of different endosymbiont isolates unconfounded by host genetic variation, but has been suggested to overestimate the realized costs of the endosymbiont in natural populations, because transfection creates new and potentially maladapted host–symbiont combinations that would be eliminated by natural selection in the field. In this experiment, we show that removing *H*.* defensa* isolates from their natural host clones with antibiotics results in a fitness gain that is comparable to the fitness loss from their introduction into two new clones. This suggests that estimating cost by transfecting endosymbiont isolates into a shared host genotype does not lead to gross overestimates of their realized costs, at least not in the two recipient genotypes used here. By comparing our data with data reported in previous publications using the same lines, we show that symbiont‐induced costs may fluctuate over time. Thus, costs estimated after extended culture in the laboratory may not always be representative of the costs at the time of collection in the field. Finally, we report the accidental observation that two isolates from a distinct haplotype of *H*.* defensa* could not be removed by cefotaxime treatment, while all isolates from two other haplotypes were readily eliminated, which is suggestive of variation in susceptibility to this antibiotic in *H*. *defensa*.

## Graphical abstract

In this experiment, we show that removing isolates of the bacterial endosymbiont *Hamiltonella* *defensa* from their natural aphid host clones with antibiotics results in a fitness gain that is comparable to the fitness loss from their introduction into new clones. This suggests that estimating costs by transfecting endosymbiont isolates into a shared host genotype does not lead to gross overestimates of their realised costs.

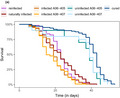

## 1 INTRODUCTION

Defensive symbiosis describes an association between two organisms that results in protection provided to the host – be it protection from enemies or environmental stress (White, 2009). Examples of defensive symbiosis can be found across different phyla: From ants or fungal endophytes that protect plants from herbivores (Clay, 1988; Janzen, 1967), to bioluminescent bacteria that allow fish to conceal their silhouette (Hastings, 1971), or bacterial endosymbionts that protect insect hosts against predators or parasitoids (Oliver & Moran, 2009). Many microbial defensive symbionts are heritable, and the evolution of their protective function has been attributed to vertical transmission, as the fitness benefit of defending a host against danger is particularly high if the symbiont's spread depends on the host's reproduction (Jones et al., 2007; Lively et al., 2005). But even if the fate of a vertically transmitted, defensive symbiont is intimately entwined with that of its host, the symbiont's presence may still be costly to the host – as long as the cost does not outweigh the benefit of the association. In its essence, symbiosis can be considered a constant battle for balance between stabilizing benefits and destructive costs (Bennett & Moran, 2015).

Identifying and quantifying costs is vital to understand how symbiosis forms and is maintained. One model organism used to investigate this are aphids. Apart from their primary endosymbiont, the γ‐proteobacterium *Buchnera aphidicola*, which supplements the aphid's diet with essential amino acids and whose removal proves fatal to the host (Douglas, 1998; Hansen & Moran, 2011; Sasaki et al., 1991), aphids associate with “secondary” bacterial endosymbionts, which substantially increase survival under specific ecological conditions. These secondary endosymbionts can, for example, defend their hosts against natural enemies (Ferrari et al., 2004; McLean et al., 2020; Oliver et al., 2003; Vorburger et al., 2009) or protect them from negative impact of heat stress (Chen et al., 2000; Russell & Moran, 2006). Given that secondary symbionts only occur at intermediate frequencies in aphid populations (Smith et al., 2015; Vorburger & Rouchet, 2016), their presence likely inflicts a cost on the host which is only offset by a benefit under specific environmental conditions (Russell & Moran, 2006; Vorburger & Gouskov, 2011).

There are basically three ways to assess the cost of secondary endosymbionts in aphids – and they all come with caveats. The simplest approach is to compare naturally infected and uninfected clones (e.g., Castañeda et al., 2010; Leybourne et al., 2020), but this confounds effects of host and symbiont genotypes. Second, one can eliminate symbionts using antibiotics and compare the fitness of cured and infected lines of the same clonal host genotype. With this approach, care must be taken to avoid lingering negative effects of the antibiotic treatment on the host or its primary endosymbiont *B*.* aphidicola*. Otherwise, there is a risk of underestimating the cost of secondary symbionts, as the cured aphid suffers from reduced fitness due to the antibiotic treatment. At the same time, this approach may not yield a general understanding of the cost of the symbiont, since it always only separates one association of host and symbiont genotypes. It is unable, for example, to account for genotype‐by‐genotype interaction between aphid clone and endosymbiont isolate (Ferrari et al., 2007; Parker et al., 2017; Vorburger & Gouskov, 2011). These sources of variation are best disentangled by transferring different isolates of a secondary endosymbiont into more than one common genetic background. However, the approach may also result in a biased perception of the costs of symbiosis. Experimental transfections create new combinations of secondary endosymbionts and aphid genotypes, similar to when aphids reproduce sexually. But unlike in nature, these new combinations created in the laboratory are not tested and optimized by natural selection. As a result, unfavorable combinations of host and symbiont genotypes, which would quickly disappear in nature, might be used to assess the costs of secondary endosymbionts. Experiments with artificial host–symbiont combinations may, thus, overestimate the realized cost of secondary endosymbionts in nature. Concerns about artificial host–symbiont pairings in aphids are known to have influenced experimental designs (Polin et al., 2014), and they were substantiated by a recent meta‐analysis of 68 papers on the effect of facultative endosymbionts on aphids. Zytynska et al. (2021) showed that – across all analyzed aphid species and secondary symbionts – the estimated costs of harboring symbionts were higher in hosts that were artificially infected with an endosymbiont than in natural host–endosymbiont associations.

In this work, we addressed these concerns using a well‐characterized set of 11 isolates of the secondary endosymbiont *Hamiltonella defensa* of the black bean aphid, *Aphis fabae* (see Cayetano et al., 2015; Vorburger & Gouskov, 2011). When these isolates were transfected into two naturally uninfected aphid clones, that is, into common genetic backgrounds, they provided protection against parasitoid wasps to *A*.* fabae* (Cayetano et al., 2015; Vorburger et al., 2009), but at the costs of reducing lifespan and offspring production in the absence of parasitoids (Cayetano et al., 2015; Vorburger et al., 2013; Vorburger & Gouskov, 2011). Mechanistically, such costs could be linked to the consumption of host resources by the symbiont, collateral damage to the host from anti‐parasitoid protection (e.g., via toxins), or from host immune activation triggered by the presence of the symbiont (Vorburger & Perlman, 2018). To test for a potential over‐estimation of costs in artificial associations, we compared the cost of these *H*.* defensa* isolates in the two naturally uninfected host genotypes, which they had been transfected into, to their cost in their naturally associated host genotypes. We also tested whether antibiotic treatment *per se* impacts the fitness of aphid clones by curing naturally infected aphid clones and reinfecting them with their own isolate of *H*. *defensa*. The two approaches allowed us to better understand the caveats for experiments with artificial host–symbiont pairings aimed at detecting the cost of symbiosis.

## 2 METHODS

### 2.1 Aphid clones and *H*.* defensa* isolates

We measured lifetime offspring production and age at death for 51 lines of *A*.* fabae* (Table S1). In Europe, *A*. *fabae* comprises several subspecies, but here we only used lines belonging to the nominal subspecies *A*. *fabae fabae*. Table S1 provides the collection information of all aphid clones and their associated *H*.* defensa*.

Of the 51 aphid lines, 11 represented clones that were collected from the field with a natural infection with *H*.* defensa* (A06‐09 to Af6, “naturally infected”). From these 11 naturally infected clones, 10 cured (A06‐09^H−^ to Af6^H−^, ‘cured’) and six cured and subsequently reinfected lines (A06‐09^H.reinf^ to AF6^H.reinf^, “reinfected”) were created. A further 12 lines each belonged to clones A06‐405 and A06‐407. These two clones were originally free of secondary endosymbionts (line 405H0, which corresponds to the treatment “uninfected A06‐405,” and line 407H0, the “uninfected A06‐407”) and had been microinjected with 11 different *H*.* defensa* isolates (H9 to HAf6) from the naturally infected *A*.* fabae* clones between 2008 and 2012 to form lines 405H15 to 405HAf6 (“infected A06‐405”) and 407H15 to 407HAf6 (“infected A06‐407”) (see Table 1 for an overview over the different aphid lines and Table S1 for creation dates of the lines; for initial generation of the lines see Cayetano et al. (2015)). Since their collection or creation, all lines have been maintained as asexually reproducing colonies on broad beans (*Vicia* *faba*) at 18–20°C and a 16 h photoperiod.

**TABLE 1:** Overview over the 51 used aphid lines used in this experiment

*H*.* defensa* haplotype	Naturally *H*.* defensa*‐infected aphid clones	Cured	Reinfected	Naturally uninfected aphid clones	Artificially infected A06‐407	Artificially infected A06‐405
–				A06‐407/407H0		
–				A06‐405/405H0		
1	A06‐76	–	–		407H76	405H76
1	A06‐101	A06‐101^H−^	–		407H101	405H101
2	A06‐09	A06‐09^H−^	A06‐09^H.reinf^		407H9	405H9
2	A06‐15	A06‐15^H−^	–		407H15	405H15
2	A08‐28	A08‐28^H−^	A08‐28^H.reinf^		407H28	405H28
2	A06‐30	A06‐30^H−^	–		407H30	405H30
2	A06‐101	A06‐101^H−^	–		407H101	405H101
2	A06‐323	A06‐323^H−^	–		407H323	405H323
2	A06‐343	A06‐343^H−^	A06‐343^H.reinf^		407H343	405H343
2	A06‐402	A06‐402^H−^	A06‐402^H.reinf^		407H402	405H402
2	Af6	Af6^H−^	Af6^H.reinf^		407HAf6	405HAf6
3	A06‐15	A06‐15^H−^	–		407H15	405H15
3	A06‐85	A06‐85^H−^	A06‐85^H.reinf^		407H85	405H85

Note

Not all aphid clones that were naturally infected with *H. defensa* could be cured from the infection, and likewise reinfection was not always successful. If a clone was naturally infected with *H. defensa*, the haplotype of *H. defensa* is indicated in the leftmost column.

Based on partial sequences of two bacterial housekeeping genes, *murE* and *accD*, the different *H*.* defensa* isolates in this experiment can be grouped into three haplotypes (Table 1, Table S1): Haplotype 1 comprises H76 and H101; haplotype 2 comprises H9, H28, H30, H323, H343, H402, and AF6; and haplotype 3 comprises H15 and H85 (Cayetano et al., 2015). The sequencing of additional genes has confirmed the division into these three haplotypes (Youn Henry, personal communication, 2020), which also possess different variants of the APSE bacteriophage (Dennis et al., 2017; Kaech et al., 2021). These phages encode different putative toxins and their presence in the *H*. *defensa* genomes is linked to the protective function of this secondary endosymbiont (Degnan & Moran, 2008; Oliver et al., 2009; Oliver & Higashi, 2019). All haplotypes protect *A. fabae* against the parasitoid wasp *Lysiphlebus fabarum*, but strength of protection varies among haplotypes, with haplotype 1 providing the strongest and haplotype 3 the weakest protection (Cayetano et al., 2015). Phylogenetically, the three *H*. *defensa* haplotypes are very distinct and more closely related to different *H*. *defensa* isolates from another aphid species (*Acyrthosiphon pisum*) than to each other (Kaech et al., 2021).

### 2.2 Curing aphid clones from *h* . *defensa*

Most aphid clones were cured from their *H*.* defensa* infection by oral uptake of the antibiotic cefotaxime (LGC Standards, Molsheim, France, C11064400). Broad bean leaves were inserted through a hole in the lid into 1.5‐ml Eppendorf tubes containing a solution of 1 mg/ml cefotaxime in tap water. Of each naturally infected aphid clone, six 3‐ to 4‐day‐old aphid nymphs were placed on the leaf. The Eppendorf tube was encased in a Falcon tube, which was sealed with a foam plug. Each clone was treated in two sequential batches, respectively, with three replicate leaves in the first and one or two leaves in the second batch. Aphids fed on the antibiotic‐laced plant sap for 48 h in the first batch and for 72 h in the second batch before being placed individually on *V*.* faba* seedlings at 18°C to reproduce. Twenty‐nine days after exposure to antibiotics, three adult daughters of the aphids that survived the antibiotic treatment were allowed to reproduce overnight on *V*.* faba* seedlings before their DNA was extracted and tested for the presence of *B. aphidicola* and *H*.* defensa*. Two aphid clones, A06‐76 and A06‐101, could not be cured with the protocol described above. We then tried to cure them in a less systematic manner. We tested different dosages of meropenem (Adooq Bioscience, Irvine, CA, USA, A10569), phosphomycin disodium (Sigma‐Aldrich, St. Louis, MO, USA, P5396), and a mixture of cefotaxime, gentamycin sulfate (Panreac Applichem, Darmstadt, Germany, A1492), and ampicillin (Calbiochem, San Diego, CA, USA, C171254) (McLean et al., 2011), applied either orally or through microinjection. Eventually, one *H*.* defensa*‐free aphid of clone A06‐101 was obtained. Its mother had been feeding for 3 days on a mixture of antibiotics (100 µg/ml ampicillin, 50 µg/ml cefotaxime, and 50 µg/ml gentamycin, dissolved in tap water). Despite extensive trials, we did not manage to cure clone A06‐76 from its *H*.* defensa* infection.

### 2.3 Reinfection of aphid clones

Approximately, 12 generations after antibiotic curing, we reinfected all cured lines with the *H*.* defensa* isolate that they originally were associated with. Four‐ to five‐day‐old aphids were injected with hemolymph from adult donors under CO_2_‐anesthesia using a FemtoJet 4i microinjector and placed in insect breeding dishes (Ø 5 cm), which contained a broad bean leaf disc (Ø 4 cm) on 1% agar. The aphids were maintained at 21°C and a 16 h photoperiod until they died. Their last three offspring were allowed to reproduce on broad bean seedlings before being tested for the presence of *H*.* defensa*. Reinfection was successful for six clones (Table S1). These were used to compare the fitness of naturally infected aphids with the fitness of cured and reinfected aphids of the same host–symbiont combination.

### 2.4 Experimental procedures

Approximately, 16 generations after the initial antibiotic treatments and four generations after reinfections, we estimated lifespan and lifetime reproduction of all 51 aphid lines (Table S1). To prevent carryover of environmental maternal effects from stock cultures, experimental setup was as follows: Two generations before the start of the experiment, 51 bean seedlings – one for each line – were infested with five adult aphids each. The adults were allowed to produce offspring for 2 days before being removed singly into Eppendorf tubes and frozen at −20°C until DNA extraction (see Molecular methods below). When the offspring had reached adulthood, they were used to setup 10 experimental blocks. Note that the 10 experimental blocks were setup in four batches (3+3+2+2) on four consecutive days. For each block, we took a potted 1‐week‐old broad bean seedling per aphid line and infested it with three adult aphids. The seedlings were covered with a cellophane bag, which was secured to the pot with a rubber band, and placed on a tray in random order (randomized complete blocks). Thus, each of the 10 blocks consisted of one tray containing one replicate of all 51 aphid lines. The trays were placed in a climatized room at 21°C and a 16 h photoperiod. The adults were allowed to reproduce overnight before being removed and discarded. At that point, the experimenter was blinded to the line identity of the replicate colonies. The experimental generation was started after 9 days, when the offspring had reached adulthood: Five reproducing adults per replicate were transferred to new bean seedlings. After 6 h, the adults were discarded and all but one offspring were removed from the plant. Then, 5 days after setup, aphids were checked for survival and transferred to new plants. Old plants were discarded. From this point onward, survival was assessed every second day and the number of offspring produced was counted every fifth day when the aphids were transferred to new plants. All aphids were followed to the end of their life. Thirteen aphids that were killed or lost during transfers were removed from the analysis.

### 2.5 Molecular methods

DNA was extracted from the 51 × 5 aphids collected two generations before the start of the experiment using a “salting out” protocol (Sunnucks & Hales, 1996). Extraction success was verified by amplifying part of the 16S rRNA gene of *B. aphidicola*, the obligate endosymbiont present in all aphids, using specific primers. The presence/absence of *H*. *defensa* was also determined by diagnostic PCR with specific primers for the same gene. Primers and cycling conditions are detailed in Table S2. Amplicons were run and visualized by capillary electrophoresis on a QIAxcel Advanced System (Qiagen AG, Hombrechtikon, Switzerland). Aphids were also genotyped at eight microsatellite loci (Coeur d'Acier et al., 2004; Sandrock et al., 2011) and allele scoring was done with GeneMarker v2.4.0. One of the five individual aphids of each line was selected at random to identify *H*. *defensa* haplotypes through amplification of *murE* and *accD* gene fragments (primers and cycling conditions in Table S2) and Sanger sequencing of the amplicon by a commercial provider (GATC Biotech AG, Köln, Germany). These checks confirmed the identity of all but one line. We found that due to an experimental error, all replicates of the aphid line 405H9 actually belonged to line 407H0. These replicates were reassigned to line 407H0 for data analysis.

### 2.6 Comparison to previous experiments

The impact of different *H*. *defensa* isolates on lifespan and reproduction of the two aphid clones A06‐405 and A06‐407 had already been assessed in Vorburger and Gouskov (2011) and Cayetano et al. (2015), which allowed a comparison of those earlier results with our present results. All three experiments were conducted in complete randomized block designs with broad bean seedlings in 0.07 L plastic pots as host plants, but each experiment was conducted in a different location and by different experimenters. Life history traits were assessed at a temperature of 21°C in this study, and at 20°C in Cayetano et al. (2015) and Vorburger and Gouskov (2011). The photoperiod was always set to 16 h, and aphids were transferred to new host plants every fourth or fifth day. In 2018, the titer of different *H*.* defensa* isolates was measured in aphids of clone A06‐407 raised at 18°C (Kaech & Vorburger, 2021). Titer was defined as the ratio of *H*.* defensa dnaK* to *A*.* fabae EF1α* copy numbers measured by qPCR.

### 2.7 Statistical analysis

Statistical analyses were done in RStudio v1.1.463 (RStudio Team, 2020) and R v3.5.1 (R Core Team, 2018) using the packages survival v3.1‐12 for survival plots (Therneau, 2020b), coxme v2.2‐16 (Therneau, 2020a) for Cox mixed‐effect models, permuco v1.1.0 for permutation of factorial ANOVAs (Frossard & Renaud, 2019), ggplot2 v3.3.2 (Wickham, 2016) and gridExtra v2.3 (Auguie, 2015) for producing Figures, and reshape2 v0.8.8 (Wickham, 2007) and plyr v1.8.6 (Wickham et al., 2020) for data wrangling.

Survival data were analyzed with a Cox mixed‐effect model testing for the effect of treatment (cured, reinfected, and naturally infected, experimentally infected (transfected) and naturally uninfected) with experimental block, clone (i.e., A06‐405, A06‐407, A06‐09, A06‐15, A08‐28, A06‐30, A06‐76, A06‐85, A06‐323, A06‐343, A06‐402, and Af6), and *H*.* defensa* isolate as random effects.

To calculate the loss of lifetime and offspring caused by *H*.* defensa*, we subtracted the lifespan of each replicate individual of infected clones from the average lifespan of all replicates of its uninfected counterpart (i.e., for each individual of clone A06‐405 infected with *H*.* defensa* isolate H101, we subtracted its lifespan from the average lifespan of all uninfected A06‐405). The isolate H76 was excluded from these analyses, as its naturally associated aphid clone, A06‐76, could not be cured from *H*.* defensa*. Since the residuals of linear mixed models deviated significantly from uniformity, we used permutation ANOVAs with the “dekker” method, which is more appropriate for unbalanced designs, and 100,000 permutations to analyze influence of genetic background (natural host clone or the two experimentally infected host clones), *H*.* defensa* isolate, the interaction between genetic background and isolate, as well as experimental block on the number of offspring and lifespan lost through infection. In two additional permutation ANOVAs, we compared lifespan and lifetime reproduction of the six reinfected aphid lines to the corresponding naturally infected lines. Treatment (reinfected or naturally infected), isolate, the interaction between treatment and isolate, as well as experimental block were treated as fixed effects.

## 3 RESULTS

### 3.1 Antibiotic treatment: failure to eliminate haplotype 1 of *H*.* defensa* with cefotaxime

Of 282 antibiotic‐treated aphids whose mothers were fed with cefotaxime‐laced plant sap, 177 (62.77%) were successfully cured of *H*.* defensa*. The success of antibiotic treatment was significantly different among *H*.* defensa* haplotypes (χ^2^ = 120.44, df = 2, *p *< .001). The percentage of cured individuals was high and very similar for aphids infected with *H*.* defensa* of haplotype 2 (78.9%) and haplotype 3 (78.2%) (χ^2^ = 0, df = 1, *p* = 1), whereas not a single individual of clones A06‐76 and A06‐101 lost the infection with *H*.* defensa*. These clones carry *H*.* defensa* haplotype 1 which we failed to remove by treatment with cefotaxime. Applying a brute force approach with multiple antibiotics at different concentrations (see Methods), we later obtained one individual of clone A06‐101 that lost its *H*.* defensa* infection, but we did not manage to cure A06‐76.

### 3.2 Costs of infection with *H*.* defensa*

**FIGURE 1:**
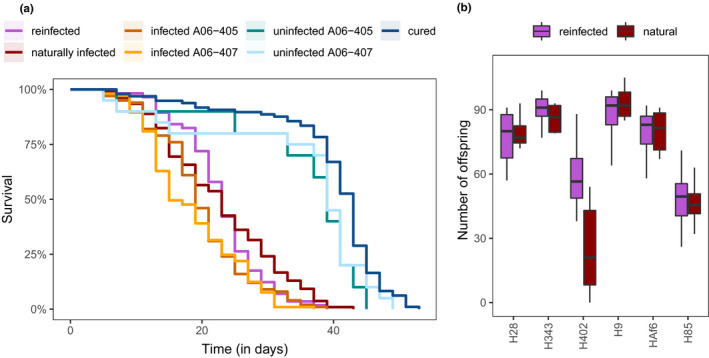
(a) Survival of aphids naturally infected by *H*.* defensa* (dark red), cured from *H*.* defensa* (dark blue), cured and reinfected (magenta), naturally uninfected (uninfected A06‐405 and A06‐407, cyan), and transfected with different *H*.* defensa* isolates (infected A06‐405 and A06‐407, orange). (b) Reproduction of aphids naturally infected by *H*.* defensa* (dark red) and aphids cured from and reinfected with *H*.* defensa* (magenta)

Of 510 aphids followed over the course of their life, 13 (2.5%) were lost due to accidents and were removed from the analysis. All aphids survived to at least the first assessment of survival at 5 days of age, with a median survival of 23 days. Different aphid lines varied in their lifespan. A Cox mixed effects model revealed a highly significant random effect of clone (χ^2^ = 15.85, df = 1, *p *< .001) and *H*.* defensa* isolate (χ^2^ = 95.83, df = 1, *p *< .001), while the random effect of the experimental block was not significant (χ^2^ = 0.05, df = 1, *p *= .817). An overview over lifespan and offspring production of all clones in the experiment is provided in Figures S1 and S2, respectively. Generally, infection with *H*.* defensa* was costly (Table 2, Figure 1a). Contrasts comparing different treatments showed that aphids infected by *H*.* defensa* died significantly earlier than uninfected aphids (Table 3). There was, however, no significant change in survival due to reinfection or between cured and naturally uninfected aphids (Table 3).

**TABLE 2:** Maximal, median, and mean longevity and reproduction in aphids that were infected with their naturally associated *H*.* defensa* isolate, cured from their infection, or cured and reinfected with their associated *H*.* defensa*, as well as longevity and reproduction in naturally uninfected aphids into which different *H*.* defensa* isolates were experimentally infected (transfected) or that were tested in their natural uninfected state

Treatment	Replicates	Age at death (in days)			Number of offspring		
		Maximum	Median	Mean	Maximum	Median	Mean
Reinfected	57	39.0	23.0	22.8	99.0	77.0	72.0
Naturally infected	108	43.0	23.0	22.7	105.0	75.5	65.7
Transfected A06‐405	100	39.0	19.0	19.7	94.0	59.5	54.3
Transfected A06‐407	105	37.0	15.0	18.3	94.0	46.0	48.0
Cured	97	53.0	43.0	39.6	106.0	88.0	82.9
naturally Uninfected A06‐405	10	45.0	39.0	35.0	86.0	80.5	73.1
Naturally uninfected A06‐407	20	49.0	39.0	34.8	96.0	68.0	60.9

Note

Treatments are sorted by infection status, with infected aphids on the top. The number of replicates in each treatment is indicated.

**TABLE 3:** Cox regression on the influence of treatment on lifespan was followed by post hoc tests with comparisons specified as custom contrasts between different experimental treatments (reinfected, naturally infected, and cured aphid clones, as well as naturally uninfected and experimentally infected (transfected) A06‐405 and A06‐407)

Post hoc comparison	Estimate	Std. error	*z* value	*p*‐value
Infected vs. uninfected lines	−3.145	0.527	−5.971	**<.001**
Reinfected vs. naturally infected lines	−0.086	0.183	−0.473	.636
Cured vs. naturally uninfected lines	0.728	1.076	0.676	.499
Naturally infected vs. transfected lines	0.804	0.456	1.763	.078

Note

Resulting *p*‐values are considered significant if they are below a *p*‐value of 0.0125, which corresponds to a Bonferroni correction.

Averaged over 10 isolates, aphids infected with *H*.* defensa* lost 16.84 offspring and their lifespan was shortened by 16.59 days compared to uninfected aphids. For a comparison between isolates see Table 4.

**TABLE 4:** Impact of different *H*.* defensa* isolates on the lifespan and reproduction of the aphid clone they were naturally associated with, and on naturally uninfected aphid clones that they were transfected into (A06‐405 and A06‐407)

*H*.* defensa* isolate	Average cost on lifespan (in days)			Average cost on reproduction		
	Naturally associated clone	Clone A06‐405	Clone A06‐407	Naturally associated clone	Clone A06‐405	Clone A06‐407
H101	17.2	6.6	13.6	28.1	5.3	4.0
H9	15.6	NA	22.0	8.5	NA	35.3
H28	12.4	17.8	15.3	0.6	20.1	−4.1
H30	7.4	13.2	14.6	−4.8	1.4	−4.6
H323	27.1	18.8	22.6	52.6	22.9	34.5
H343	15.8	14.0	20.2	0.9	−0.9	23.1
H402	28.2	17.2	12.4	60.6	18.2	−3.4
HAf6	11.6	14.6	21.2	−0.7	36.7	28.2
H15	8.2	7.6	8.2	4.7	−5.9	−14.57
H85	30.6	23.6	25.0	39.3	56.2	48.6

Note

Costs are averaged over all replicates and expressed in days of life and number of offspring lost (or gained, if there is a negative value) due to infection with *H. defensa*. Missing data, caused by failure to include line 405H9 into the experiment, are indicated by “NA.” Aphids infected with isolate H76 are not included due to inability to cure the A06‐76 line.

The amount of lifespan lost depended significantly on the *H*. *defensa* isolate (*F*_9,247_ = 29.14, *p*_permutation_ < .001) but not on whether the isolate was associated with its natural aphid genetic background or experimentally transferred to one of the originally uninfected clones (*F*_2,247_ = 0.01, *p*_permutation_ = .869). However, there was a significant interaction between genetic background and *H*.* defensa* isolate (*F*_18,247_ = 6.53, *p*_permutation_ < .001), indicating that the impact of different *H*. *defensa* isolates varied significantly depending on which aphid clones they were associated with. Lifespan did not vary between experimental blocks (*F*_9,247_ = 0.96, *p*_permutation_ = .475). Similarly, the number of offspring lost depended significantly on *H*.* defensa* isolate (*F*_9,247_ = 21.41, *p*_permutation_ < .001) and the interaction between isolate and background (*F*_18,247_ = 7.00, *p*_permutation_ < .001), but there was no significant main effect of genetic background (*F*_2,247_ = 1.07, *p*_permutation_ = .146) or experimental block (*F*_9,247_ = 0.97, *p*_permutation_ = .464).

### 3.3 Natural infections versus reinfections: no negative effect of antibiotic treatment

A separate analysis of six clones, for which naturally infected as well as cured and reinfected lines were available, provided no evidence that the antibiotic curing has any long‐lasting negative effects on aphid fitness. The clones varied significantly in average lifetime reproduction and lifespan, and there was a significant or near‐significant overall difference between naturally infected and reinfected lines (Table 5). There was also a significant interaction between aphid clone and treatment (natural or reinfected) for reproduction and lifespan (Table 5). The significant main effect of treatment and the significant interaction between treatment and isolate were driven exclusively by clone A06‐402 (Figure 1b). Its naturally infected line exhibited conspicuously low fitness, which was improved in the cured and reinfected line. When clone A06‐402 was excluded from the analysis, the among‐clone variation remained significant, but there was no significant effect of treatment and no clone‐by‐treatment interaction (Table 5).

**TABLE 5:** Results of permutation ANOVAs assessing the influence of antibiotic cure with subsequent reinfection on aphid fitness

Source	All clones			Clone A06‐402 excluded		
	df	*F*	*p_perm_*	df	*F*	*p_perm_*
(a) Lifespan						
Experimental block	9	0.57	.835	9	0.49	.901
Treatment (natural vs. reinfected)	1	3.77	.054	1	0.01	.910
Isolate	5	26.65	**<.001**	4	30.74	**<.001**
Treatment × isolate	5	4.91	**<.001**	4	0.70	.609
(b) Lifetime reproduction						
Experimental block	9	1.22	.285	9	1.30	.236
Treatment (natural vs. reinfected)	1	5.81	.**016**	1	0.39	.544
Isolate	5	28.21	**<.001**	4	20.21	**<.001**
Treatment × isolate	5	3.49	.**005**	4	0.50	.752

Note

Response variables were (a) lifespan or (b) lifetime reproduction. Predictors were treatment (natural or reinfected), *H. defensa* isolate, and experimental block. The models were run with or without clone A06‐402. *p*‐values are based on the permutation method “dekker” with 100,000 permutations.

### 3.4 Costs of *H*.* defensa* changed over time

**FIGURE 2:**
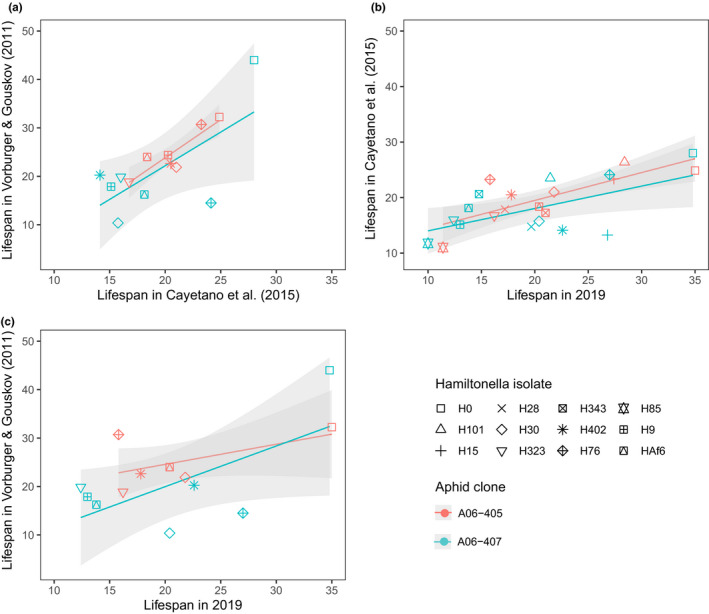
Lifespan of aphid clones A06‐405 (orange) and A06‐407 (blue) with or without *H*.* defensa*‐infection compared among three different experiments. Shapes indicate absence of *H*.* defensa* (H0) or infection with *H*.* defensa* (isolates H15 to HAf6). (a) Pearson's product‐moment correlation for A06‐405 (*r* = .89, *t*_5_ = 4.5, *p *= .006) and for A06‐407 (*r* = .67, *t*_5_ = 2.01, *p *= .101) between Vorburger and Gouskov (2011) and Cayetano et al. (2015). (b) Pearson's product‐moment correlation for A06‐405 (*r* = .77, *t*_9_ = 3.64, *p *= .005) and for A06‐407 (*r* = .59, *t*_10_ = 2.30, *p *= .044) between Cayetano et al. (2015) and this study. (c) Pearson's product‐moment correlation for A06‐405 (*r* = .56, *t*_4_ = 1.37, *p *= .243) and for A06‐407 (*r* = .64, *t*_5_ = 1.87, *p *= .121) between Vorburger and Gouskov (2011) and this study

**FIGURE 3:**
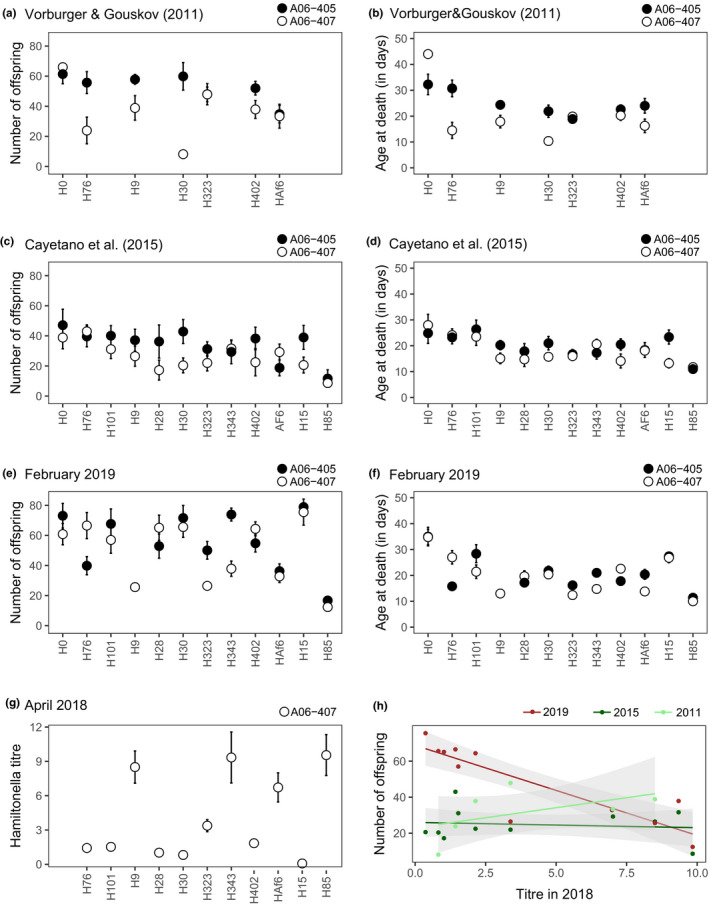
Costs of different *H*.* defensa* isolates varied between 2011 and 2019. The two aphid clones A06‐405 (black) and A06‐407 (white) have been maintained in clonal lines either uninfected with *H*. *defensa* (H0) or infected with different *H*.* defensa* isolates (H16 to H85). Lifetime reproduction of the lines was measured in (a) 2011, (c) 2015, and (e) 2019; and lifespan was measured in (b) 2011, (d) 2015, and (f) 2019. (g) Titer, defined as the ratio of *H*.* defensa dnaK* to *A*.* fabae EF1α* copy numbers, was measured for *H*.* defensa* isolates in clone A06‐407 in 2018 by Kaech and Vorburger (2021). (h) Titer of different *H*.* defensa* isolates in A06‐407 compared to reproduction of *H*.* defensa*‐infected A06‐407 in 2011, 2015, and 2019. Error bars indicate standard errors

The *H*.* defensa*‐infected and uninfected lines of aphid clones A06‐405 and A06‐407 have been maintained clonally in laboratory settings for approximately a decade. Their life history traits have previously been assessed by Cayetano et al. (2015) and Vorburger and Gouskov (2011). This provided an opportunity to examine the consistency of *H*.* defensa*‐induced costs over many years of laboratory culture. Generally, lifespan (Figure 2) and lifetime reproduction (Figure S3) correlated positively across studies, with the two experiments conducted at 20°C (2015 and 2011) correlating more with each other than with the data from this experiment, which was conducted at 21°C. Despite these overall correlations, costs of different *H*.* defensa* isolates on their host's reproduction (Figure 3a,c,e) and lifespan (Figure 3b,d,f) varied considerably over time. In A06‐407, for example, costs of isolate H30 on both lifespan and reproduction decreased successively, while costs of H323 on reproduction increased over time.

In 2018, the titer of different *H*.* defensa* isolates was measured in clone A06‐407 (Figure 3g; data from Kaech and Vorburger (2021)). The *H*.* defensa* titers estimated in 2018 show a negative correlation with the number of offspring produced by *H*.* defensa*‐infected A06‐407 in 2019 (Pearson's product‐moment correlation: *r* = −.87, *t*_9_ = −5.35, *p *< .001) and with lifespan in 2019 (*r* = −.82, *t*_9_ = −4.28, *p *= .002). Titer does, however, not correlate with fitness components measured in 2015 and 2011 (Figure 3h), neither for reproduction (2015: *r* = −.12, *t*_9_ = −0.37, *p *= .717 and 2011: *r* = .50, *t*_4_ = 1.17, *p *= .308) nor for lifespan (2015: *r* = −.13, *t*_9_ = −0.40, *p *= .696 and 2011: *r* = .34, *t*_4_ = 0.77, *p *= .486; Figure S4).

## 4 DISCUSSION

In novel pairings of hosts and endosymbionts, initial lack of adaptation has repeatedly been shown to manifest in significant costs to the host, which may get alleviated during subsequent adaptation between host and symbiont – for example, in *Wolbachia pipientis* and the parasitoid wasp *Nasonia* (Chafee et al., 2011), *Spiroplasma* and *Drosophila* (Nakayama et al., 2015), or *Burkholderia agricolaris* and the amoeba *Dictyostelium discoideum* (Shu et al., 2018). Murfin et al. (2015) showed in *Steinernema* nematodes and their bacterial symbiont *Xenorhabdus bovienii* that costs of infection in new host–symbiont pairings are lower when the phylogenetic distance between the old and the new symbiont is small. This implies that costs of infection with an endosymbiont may be lower in natural associations tested by natural selection than in artificial associations generated experimentally. However, Russell and Moran (2005) moved some aphid symbionts between species with little obvious cost to the new host, and it is not clear how costs of infection manifest when an endosymbiont is not moved between species but among genotypes of a single species. It is conceivable that endosymbionts of sexually reproducing hosts have evolved to be tolerant to changes in host genotype to avoid fitness loss from genetic incompatibilities.

Here, we transferred multiple isolates of the defensive endosymbiont *H*.* defensa*, whose *A. fabae* host reproduces sexually once a year (Sandrock et al., 2011), to two new host genotypes. Although there were genotype‐by‐genotype interactions between aphid clones and endosymbiont isolates as previously described (Chong & Moran, 2016; Vorburger & Gouskov, 2011), we show that – over all *H*.* defensa* isolates – costs did not differ significantly between natural and experimental combinations. This seems to indicate that estimating costs of infection with *H*.* defensa* after artificial introduction into a common genetic background does not systematically overestimate costs in comparison to natural infections. An important caveat is, however, that we evaluated experimental infections in only two genetic backgrounds. Likely these two clones were not fully representative of the average susceptibility of *A*.* fabae* to infection with *H*.* defensa*. Our findings will have to be generalized by comparing natural and artificial pairings in more clones. Thus, it is still important to consider potential differences between natural and artificial host–endosymbiont pairings when planning and evaluating experiments. Secondly, the life‐history costs of symbiosis that we measured in this experiment correspond to only a subset of the costs an aphid host is expected to experience in the wild. We might also have to consider additional, ecological costs, such as reduced defensive behavior entailing higher susceptibility to predators (Dion et al., 2011; Polin et al., 2014) or altered interactions with other mutualists like ants (Hertaeg et al., 2021), to fully understand the impact of changes in host genotype on the cost of symbiosis. Finally, our conclusion hinges on the assumption that curing aphids of *H*.* defensa* with an antibiotic (cefotaxime) does not have any lasting (multigenerational) effects on the aphids, for example, by harming the primary endosymbiont *B*.* aphidicola*. Such an effect would compromise the fitness of the cured lines and lead to an underestimation of costs of natural infections. This assumption was addressed, and although we observed aphids that did not reproduce immediately after cefotaxime treatment, the comparison of naturally infected with cured and reinfected lines approximately 16 generations after antibiotic exposure indicates that antibiotics do not inflict persistent damage. Thus, experiments assessing costs of *H*. *defensa* in naturally infected and cured aphids should not suffer from bias if the aphids are allowed to recover from antibiotic exposure.

An interesting and unexpected observation was that the two *H*. *defensa* isolates belonging to haplotype 1 could not be removed from aphids with the same antibiotic treatment (cefotaxime) that reliably eliminated all other isolates. We cannot exclude that these two aphid clones happened to be less prone to take up antibiotics, for example, by feeding less, but since both of them carried haplotype 1 *H*. *defensa*, it appears more likely that this haplotype is less susceptible to cefotaxime. Whether these symbionts are more resistant to the antibiotic because of their physical location in the host, for example, a more sheltered, predominantly intracellular lifestyle (comparable to *B*.* aphidicola*), or because they possess some genetic form of resistance, remains to be investigated. But it is important to know for future work that there may be among‐strain variation in *H*. *defensa* for the susceptibility to antibiotics.

Another surprising observation was the case of clone A06‐402, for which the cured and reinfected line showed improved survival and reproduction compared to the original, naturally infected line. Potentially, this aphid clone might have possessed two strains of *H*.* defensa*, of which only one – which happened to be less costly – was transferred at reinfection. We do not consider this likely as we have no evidence for a double infection, but with strain typing by Sanger sequencing it would be possible to miss a less common variant. Alternatively, it is also possible that the naturally infected aphid line suffered from another, opportunistic infection at the time when the experiment took place (see below), especially given that its fitness was conspicuously low. This brings us to a more general concern, the apparent fluctuations in symbiont‐induced phenotypes we observed over time. How representative are the costs of infection with *H*.* defensa* estimated here of the costs at the time when the aphids and symbionts were collected in the field? After all, some isolates used in this experiment have been associated with their natural and experimental aphid partners for approximately a decade of laboratory culture. This is a much longer association than those in natural populations, where sexual reproduction generates new combinations of host and symbiont genotypes every year. Additionally, only a small number of aphids are used to found each subsequent generation in laboratory culture. This likely allows drift to determine the aphid lines' evolution. Long‐term association between host and endosymbiont and relaxed competition between hosts under the benign laboratory conditions might facilitate the evolution of selfish endosymbionts that become more costly (Bennett & Moran, 2015; Stoy et al., 2020). Yet, comparison of the costs that *H*.* defensa* inflicts in long‐term artificial association with clones A06‐405 and A06‐407 revealed no clear trend toward increasing costs. Instead, some isolates seemed to follow independent trajectories toward increasing or decreasing costs in a way that is difficult to explain by environmental variation (different experimenters, different temperatures, or potentially different humidity). Since decreased host fitness has already been connected to high endosymbiont titers in a number of insect endosymbionts (Chong & Moran, 2016; Chrostek & Teixeira, 2015; Herren et al., 2014; Mathé‐Hubert et al., 2019), including *H*. *defensa* (Weldon et al., 2013), we compared fitness costs to *H*. *defensa* titer. Only the costs imposed by different isolates in 2019 correlated well with *H*.* defensa* titers assessed in 2018. We, thus, suspect that the temporal variation in costs might be related to changes in symbiont population sizes, potentially caused by fluctuations in *H*.* defensa's* virulence, changes in its interaction with the host's immune system, or simply drift, which also appears to induce temporal variation of *Wolbachia* load in captive lines of *Drosophila melanogaster* (Bénard et al., 2021). Another possible explanation for the temporal variation in costs is that laboratory aphid lines may get infected occasionally by other pathogens, which affect lifespan or reduce reproduction. Since our PCR primers target specific symbionts, we had no means of detecting invasion of pathogens such as viruses, fungi, or even gut bacteria into the aphids' microbiome. The undetected presence of opportunistic pathogens would both explain the apparent changes in costs of host‐isolate associations as well as the recovery of the fitness of aphid clone A06‐402 after antibiotic exposure and subsequent reinfection with its own *H*.* defensa* isolate.

In conclusion, our experiment indicates that in the absence of parasitoids, the fitness gain of losing a natural infection with *H*. *defensa* is comparable to the fitness loss from acquiring a new infection experimentally in black bean aphids. This indicates that assessing costs in a common host genetic background should be a valid strategy. However, we only tested experimental associations with two recipient clones – further studies using a more diverse set of clones are needed to generalize these results. Additionally, the apparent instability of costs induced by different *H*. *defensa* isolates over time casts doubt on whether assessment in the laboratory after long‐term laboratory culture is representative of the situation in the field. In the present case, both naturally infected and experimentally infected lines had been in long‐term culture, hopefully precluding bias, but it appears that the longer aphids are maintained in clonal cultures, the more the host–endosymbiont relationship may change. The reasons for the apparent temporal instability of *H*.* defensa*‐induced costs to the host warrant further investigation, and they should be a consideration in experiments with lines that have been in laboratory culture for extended periods of time.

## CONFLICT OF INTERESTS

The authors declare no competing interests.

## AUTHOR CONTRIBUTIONS

**Heidi Kaech:** Conceptualization (equal); investigation (lead); writing – original draft (lead); writing – review & editing (equal). **Stephanie Jud:** Conceptualization (supporting); investigation (supporting); writing – review & editing (equal). **Christoph Vorburger:** Conceptualization (equal); supervision (lead); writing – review & editing (equal).

## Supporting information

Fig S1‐S4

Table S1‐S2

## Data Availability

The data have been provided to Dryad Digital Repository and can be accessed at https://doi.org/10.5061/dryad.wpzgmsbmz.
