# Supplementary material for: Similar cost of Hamiltonella defensa in experimental and natural aphid‐endosymbiont associations
Source: Ecol Evol. 2022 Jan 24;12(1):e8551. doi: 10.1002/ece3.8551 (PMC8796928; doi:10.1002/ece3.8551)
Supplement: Supplementary file 1 — Fig S1‐S4 [file ECE3-12-e8551-s001.pdf]

Supplementary figures

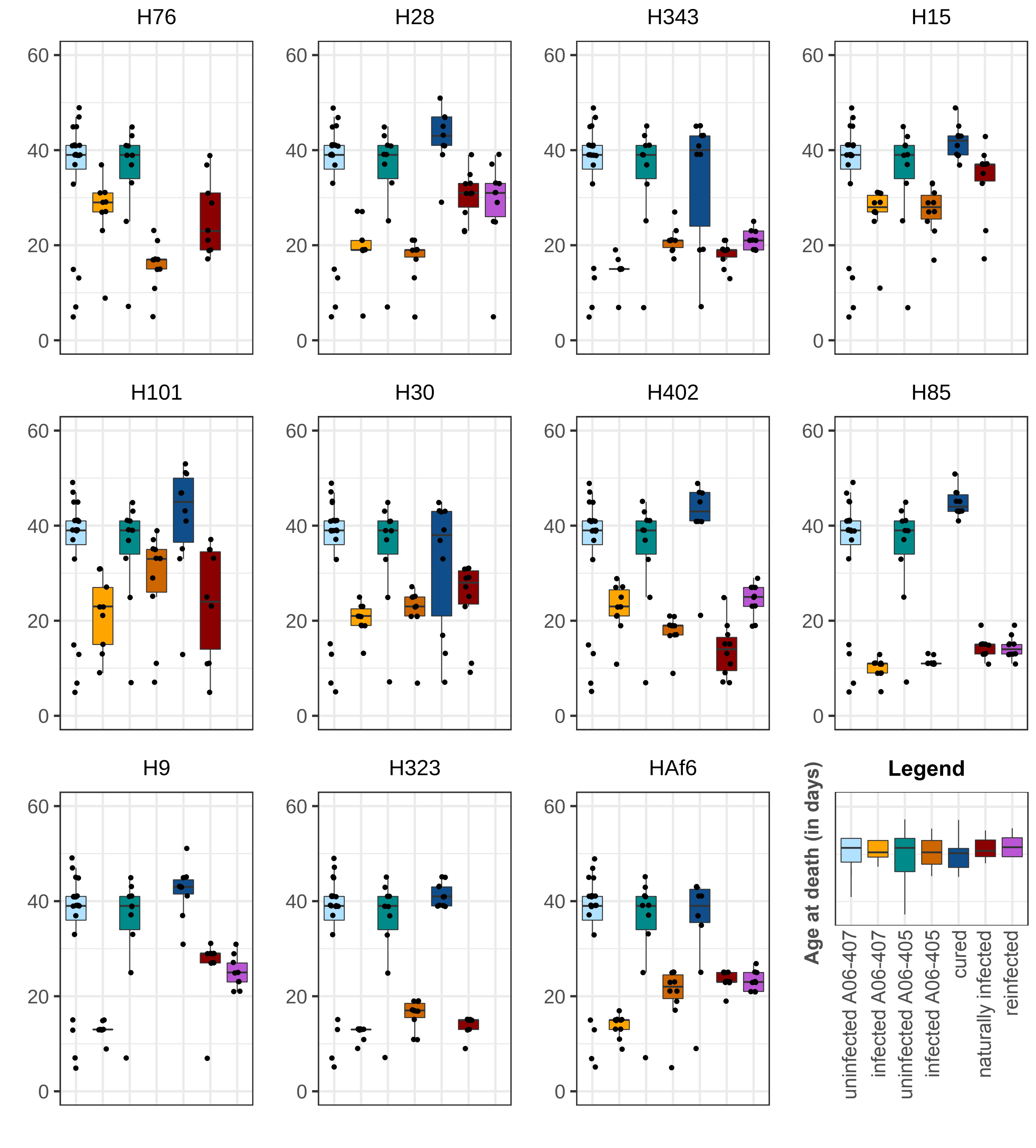

Supplementary Figure 1 – Variation in lifespan depends on infection status. Age at death for all *A. fabae* clones and sublines infected with a specific *H. defensa* isolate. The legend is in the lower right corner. Light blue and turquoise indicate lifespan of the naturally uninfected aphid clones A06-407 and A06-405, respectively. Dark yellow and orange indicate lifespan of A06-407 and A06-405, respectively, when infected with the *H. defensa* isolate. Dark blue, dark red and magenta indicate lifespan of the cured, naturally infected and reinfected clone that the *H. defensa* was associated with in nature.

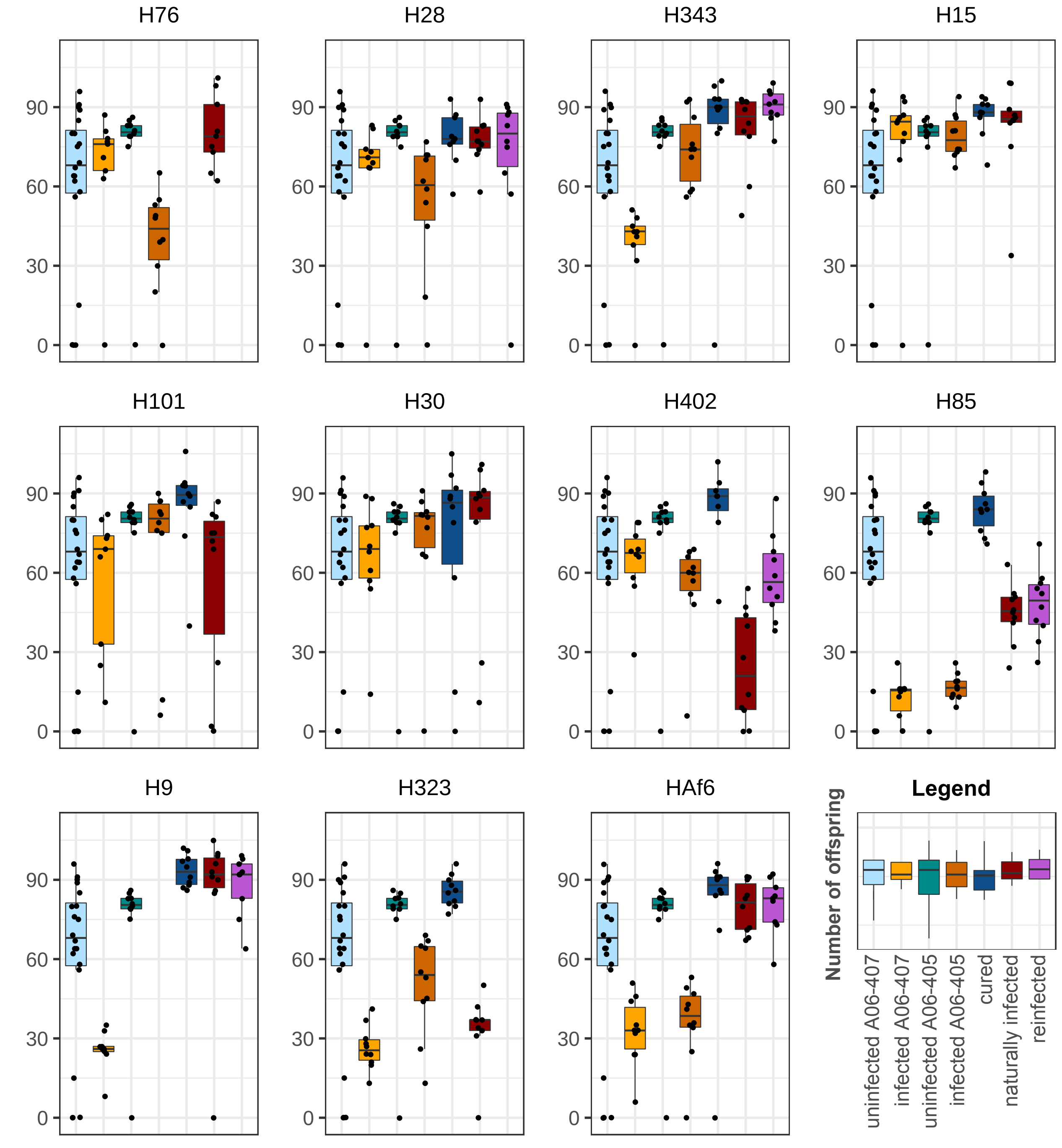

Supplementary Figure 2 – Variation in lifetime reproduction depends on infection status. Number of offspring produced over their entire lifespan for all *A. fabae* clones and sublines infected with a specific *H. defensa* isolate. The legend is in the lower right corner. Light blue and turquoise indicate reproduction of the naturally uninfected aphid clones A06-407 and A06-405, respectively. Dark yellow and orange indicate reproduction of A06-407 and A06-405, respectively, when infected with the *H. defensa* isolate. Dark blue, dark red and magenta indicate reproduction of the cured, naturally infected and reinfected clone that the *H. defensa* was associated with in nature.

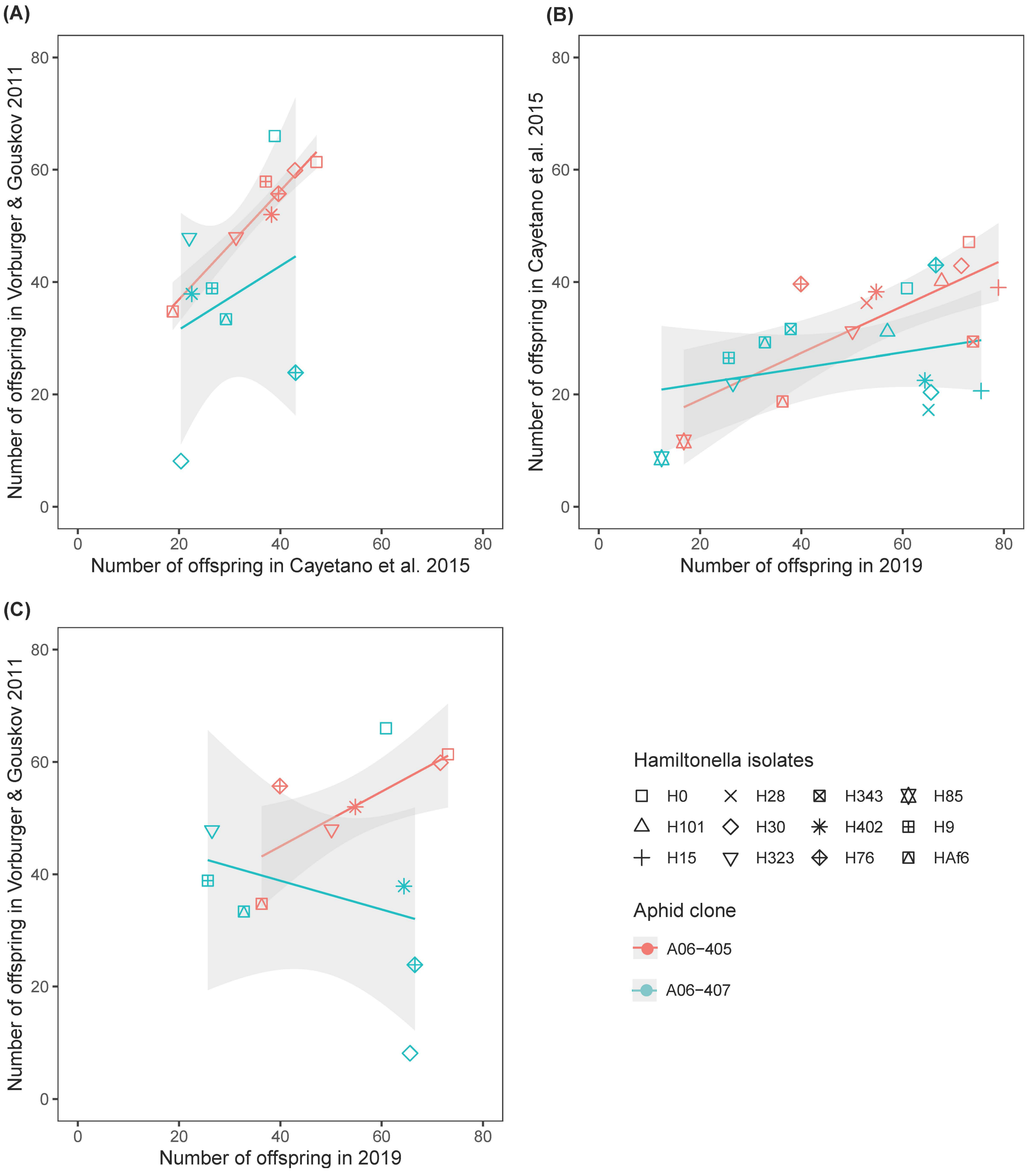

Supplementary Figure 3 – Number of offspring of aphid clones A06-405 (orange) and A06-407 (blue) with or without H. *defensa*-infection compared among three different experiments. Shapes indicate absence of *H. defensa* (H0) or infection with *H. defensa* (strains H15 to HAf6). (A) Pearson's product-moment correlation for A06-405 (r=0.97, t_5=8.80, p<0.001) and for A06-407 (r=0.27, t_5=0.64, p=0.551) between Vorburger and Gouskov (2011) and Cayetano et al. (2015). (B) Pearson's product-moment correlation for A06-405 (r=0.76, t_9=3.47, p=0.007) and for A06-407 (r=0.30, t_{10}=1.01, p=0.337) between Cayetano et al. (2015) and this study. (C) Pearson's product-moment correlation for A06-405 (r=0.77, t_4=2.45, p=0.071) and for A06-407 (r=0.27, t_5=-0.64, p=0.551) between Vorburger and Gouskov (2011) and this study.

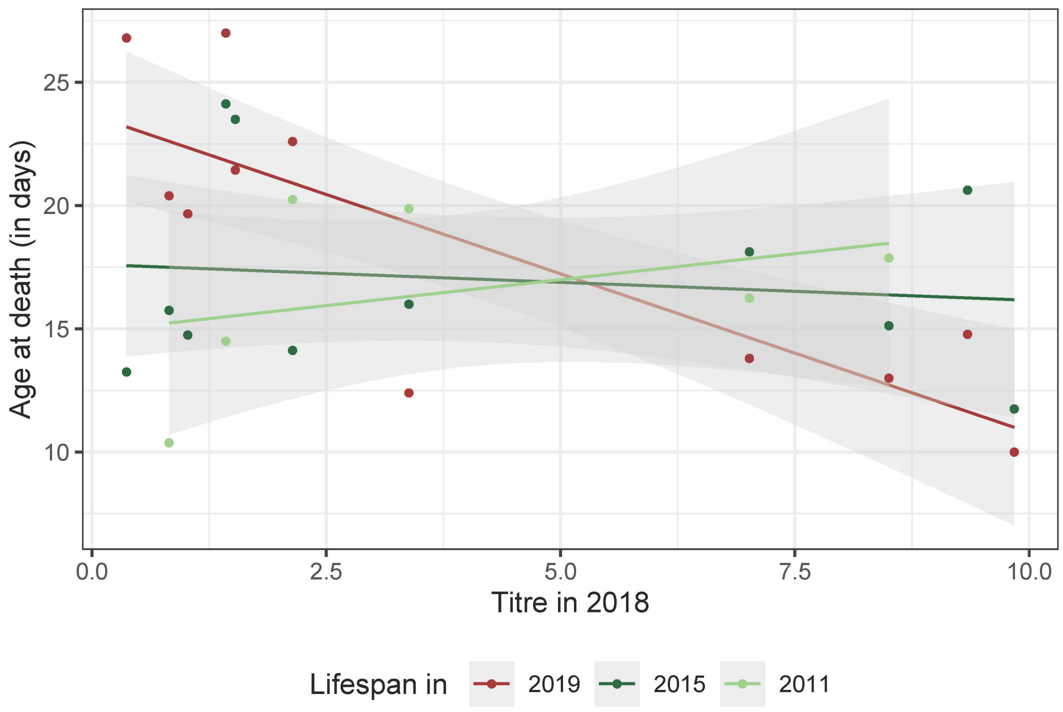

Supplementary Figure 4 – Titre of different *H. defensa* isolates in A06-407 (in 2018) compared to lifespan of *H. defensa*-infected A06-407 in 2011, 2015 and 2019.
